# Toxicity of co-exposure of microplastics and lead in African catfish (*Clarias gariepinus*)

**DOI:** 10.3389/fvets.2023.1279382

**Published:** 2023-10-06

**Authors:** Hamdy A. M. Soliman, Sally M. Salaah, Mohamed Hamed, Alaa El-Din H. Sayed

**Affiliations:** ^1^Department of Zoology, Faculty of Science, Sohag University, Sohag, Egypt; ^2^Fresh Water Division, National Institute of Oceanography and Fisheries, NIOF, Alexandria, Egypt; ^3^Department of Zoology, Faculty of Science, Al-Azhar University (Assiut Branch), Assiut, Egypt; ^4^Department of Zoology, Faculty of Science, Assiut University, Assiut, Egypt; ^5^Molecular Biology Research and Studies Institute, Assiut University, Assiut, Egypt

**Keywords:** fish, polyethylene, lead, antioxidants, cytokines, electrolyte

## Abstract

Microplastics (MPs) are an emerging threat to freshwater ecosystems with several ecotoxicological ramifications for fish. Microplastics (MPs) can adsorb heavy metals on their surfaces and increase their availability to aquatic organisms. The combined impact of lead and microplastics on fish has only been studied seldom utilizing a variety of markers. The present study aimed to evaluate the hematological, biochemical, and inflammatory signals (cytokines), as well as antioxidant enzymes in African catfish (*Clarias gariepinus*) exposed to lead (Pb) and MPs individually and combined for 15 days (acute toxicity experiment). The fish were split into four groups, the first of which was the control group. The second group received exposure to 1 mg/L of lead nitrate [Pb(NO_3_)^2^]. The third group was given 100 mg/L of MPs. A solution containing 100 mg/L of MPs and 1 mg/L of lead nitrate [Pb(NO_3_)^2^] was administered to the fourth group (the combination group). According to the findings, when MPs and Pb were combined for 15 days, the red blood cells (RBCs), thrombocytes, and lymphocytes were significantly reduced in comparison to the control fish. When compared to the control fish, the fish exposed to MPs and Pb alone or together showed a significant rise in blood interleukin-1β (IL-1β) and interleukin-6 (IL-6) cytokines. Both MPs and Pb exposure in catfish resulted in significant changes in the plasma electrolytes. The fish treated with MPs and Pb individually or in combination showed significant reduction in superoxide dismutase (SOD) and total antioxidant capacity (TAC) levels compared to the control group. The fish exposed to the combined action of MPs and Pb showed a considerable modification in all biochemical markers. The difference in the mean concentration of Pb (mg/L) between the fish exposed to Pb alone and the fish subjected to Pb and MPs combination was not statistically significant. In conclusion, according to this investigation, exposure to Pb caused an insignificant increase in Pb accumulation when MPs were present. However, co-exposure may result in anemia, cellular harm, extremely high levels of oxidative stress, and an inflammatory reaction.

## 1. Introduction

Microplastic contamination has become a global issue as a result of industrialization and the increasing usage of plastic products. By 2030, it is projected that 53.000 million kilograms of plastic will have been manufactured globally (1). The discovery of plastic dominance in macro-debris demonstrated that the rise in plastic consumption will be consistent with plastic pollution (2, 3). The macroplastics will inevitably break down and fragment to produce subsequent microplastics (secondary microplastics) (4). The major microplastics are the other type (primary microplastics) which are purposefully made small and are commonly utilized as a cleaner for self-hygiene products (5). Microplastics are found everywhere, from the surface of the earth to the open ocean. The majority of terrestrial microplastics come from residential effluent (6), which drains into the sea and rivers. Secondary microplastic production from terrestrial trash is approximately 22 folds greater than that from marine garbage (7). Estuaries and coastal environments are being heavily impacted by anthropogenic pressure in numerous regions of the world because of complex mixes of persistent organic pollutants (POPs), heavy metals, other known and undiscovered substances, and microplastics (8). Microplastics and heavy metals interact more easily since they both enter the aquatic environment through the same channels (wastewater discharges, rivers, or urban runoff) (9). Because MPs can modulate the utilization and toxicity of heavy metals through desorption mechanisms, their contact with plastic particles can be troublesome (10). As a result, it is concerning when MPs and heavy metals are present in ecosystems close to human activities (such as in mining sites) (11). When genuine and used PE particles were used to adsorb various metal ions in freshwater, it was found that the main factor influencing adsorption was the difference in ion concentration between the liquid phase (metal solution) and the solid phase (PE particles), and the metal ion concentration eventually reached equilibrium (12). Due to oxidation and weathering, the surface shape of aging particles changed, making it simple to assemble an electric charge and adsorb metal ions to establish charge balance (13). Moreover, the period that microplastics have been present in the fresh and marine water has a major effect on the adsorption capacity in addition to pH. Additionally, Brennecke et al. (14) attested the binding of fresh PS pellets and aged PVC pieces to several heavy metals during the course of 14 days of experimental manipulation. On the surface of the plastic particles, there were 800 times more heavy metals than there are in saltwater. The particular surface area of the aged PVC fragments increased as a result of cracking and crushing, and the presence of attaching biomass on the surface of the particles boosted the behavior of adsorbing heavy metals.

Our earlier studies have shown that fish exposed to microplastic frequently experience adverse physiological and biochemical impacts, gastrointestinal scratches and obstructions, behavioral issues, histopathological changes, decreased mineral integration, neurotoxicity, reproduction disorder, and even mortality (15–24). Fish can be harmed by additives found in microplastics such as polybrominated diphenyl ethers (PBDE), bisphenol A (BPA), nonylphenol (NP), and octylphenol (OP). Ingesting plastic additives can harm fish organs like the liver, kidney, and intestine through oxidative stress, physical damage, and inflammation (25).

The consequences of these interactions between heavy metals and microplastics can be antagonistic or synergistic effects (26). Although this is the case, classical threat evaluation has frequently focused on single-exposure investigation, which has resulted in an under- or overestimation of the potential harm that toxins may pose to aquatic life (27). Hence, assessing the interactions between various pollutants is particularly crucial for risk assessment. Khan et al. (28) observed that *Danio rerio* exposed to MPs that were treated with silver (Ag) had a higher fraction of intestinal Ag. Also, Luís et al. (8) found that the existence of microplastics in the water affected the immediate impact of Cr (VI) on early juvenile goby by reducing hunting efficiency and increasing fish oxidative harm. In the seabass, *Dicentrarchus labrax*, microplastics and mercury alone and in combination led to neurological damage, oxidative stress in the brain and muscle, and altered energy-related enzyme activity (29). In zebrafish, *Danio rerio*, polystyrene (PS) MPs promote cadmium accumulation (30); however, they have the reverse impact in discus fish (*Symphysodonae quifasciatus*) (31). According to a different study, greater polystyrene microplastic levels raise the toxicity of cadmium in zebrafish larvae while lower polystyrene microplastic levels reduce it (32). In the instance of copper, polyethylene (PE) MPs enhance Cu-induced oxidative harm and DNA deterioration in *Prochilodus lineatus* while having little effect on hepatic copper buildup (33). However, it has been found that concurrent exposure of *Danio rerio* larvae to copper and microplastic polymers causes neurological damage, alters behavioral patterns, and impairs growth and longevity (11, 34, 35). Additionally, polyvinyl chloride microplastic acts as a copper carrier, encouraging Cu accumulation in the liver of common carp (*Cyprinus carpio*) and intensifying inflammation (36). Zheng et al. (37) found that in the initial phases of exposed *Danio rerio* and their offspring that were not exposed, particulates rather than Zinc ions produced by ZnO NPs increased MPs toxicity.

Lead [Pb(II)] pollution of aquatic habitats is widespread as a result of the release of industrial lead-containing sewage, the accumulation of lead-containing airborne particles, and other factors (38). Several authors have reported on the toxicity of lead nitrate in fish (20, 39, 40).

Because of its vast range, ease of availability, and susceptibility to xenobiotics, catfish (*Clarias gariepinus*) are frequently utilized as fish bio-indicators (41). Furthermore, because of its all-devouring eating habits, tendency to live in sediment, and potential exposure to microplastics that could aggravate metal and pollutant accumulation, this species tends to accumulate more heavy metals (42).

In natural water bodies, the coexistence of MPs and Pb(II) is frequent and can affect the co-toxicity and migratory behavior of contaminants (43, 44). Little is known about the harmful consequences brought on by the combination of lead and microplastics in fish (45, 46).

The current study’s objective was to use various biomarkers (hematological, biochemical, and inflammatory signals (cytokines), as well as antioxidant enzymes) to examine the harmful effects of lead and microplastic exposures on catfish both singly and in combination.

## 2. Materials and methods

### 2.1. Chemicals

Toxemerge Pty Ltd. was where the MPs powder was purchased (Melbourne, Australia). A stock solution was created from the powder per the manufacturer’s instructions using filtered water (Milli-Q) and stored at 4°C in the dark. Before each usage, the stock solution (2.5 g MP/L) was sonicated. From this stock, more dilutions were made right away whenever the rising water was replaced. At Sigma-Aldrich, lead nitrate [Pb (NO_3_)_2_] was bought (St. Louis, Missouri, United States). The fresh stock solution included 1,000 mg of lead nitrate per liter of deionized water.

### 2.2. Fish exposure

The Fish Biology and Pollution Laboratory in the Faculty of Science, Assuit University, received African catfish (*C. gariepinus*; weight 250–300 g; length 20–25 cm) from an aquaculture farm. The fish were healthy and free of parasites, per AFS-FHS, 2007. The fish were put in 100 L tanks with dechlorinated tap water and air pumps, the fish were acclimated for 2 weeks in a lab setting. Conductivity 5.8 ms/cm, pH 7.2, dissolved oxygen 8.6 mg L^−1^, temperature 27.5°C, and photoperiod 12:12 light: dark were the physicochemical characteristics of the rearing water.

For each treatment, the fish were split into four groups, 30 fish for each group in triplicate. The first group was the control group. The second group was subjected to 1 mg/L Lead nitrate [Pb (NO_3_)^2^] according to Hamed et al. (22). The third group was subjected to 100 mg/L MPs according to Hamed et al. (20). The fourth group (the combination group) was subjected to a solution containing a combination of 100 mg/L of MPs and 1 mg/L of lead nitrate [Pb(NO_3_)^2^]. After 15 days, blood was drawn from the caudal vein of six randomly chosen fish from each group, whose anesthesia had been administered with ice in order to reduce dissection strain (20, 22). The blood was then analyzed for lead concentration, hematological and biochemical parameters, as well as blood electrolytes concentration, antioxidant enzymes, and inflammatory signals (cytokines).

### 2.3. Hematological indices

According to Fazio (47), various hematological indices such as red blood cells (RBC’s)and white blood cells [WBC’s] count; Differential WBC’s; blood Platelets; Hematocrit level (Hct), Hemoglobin level (Hb); Erythrocyte indices including mean corpuscular hemoglobin (MCH), Mean corpuscular volume (MCV), and mean corpuscular hemoglobin concentration (MCHC), were determined by using automated technical analyzer (BC-2800 from Mindray).

### 2.4. Inflammatory signals (cytokines)

The cytokines (Interleukin-6 and Interleukin-1β) were measured in the serum using commercially available, highly sensitive ELISA kits (Human Ultrasensitive, BioSource International Inc.).

### 2.5. Blood electrolytes

With the aid of atomic absorption spectroscopy (Model SensAA G3000, GBC Scientific Equipment Pty Ltd., Dandenong, Victoria 3175, Australia), blood electrolytes (HCO_3_, Na^+^, K^+^, Cl^−^, Fe^+2^, and Ca^+2^) were determined in serum (48). The wavelengths of the elemental ions were chosen to have the proper sensitivity for the concentration range.

### 2.6. Antioxidant parameters and Lipid peroxidation

The activities of superoxide dismutase (SOD), total antioxidant capacity (TAC), and glutathione S-transferase (GST) were measured in serum samples using the methods of Koracevic et al., Nishikimi et al., and Sayed et al. (49–51), respectively. The malondialdehyde (MDA) level was determined using a thiobarbituric acid reaction (52).

### 2.7. Biochemical parameters

According to Bricknell et al. (53), the blood was spun for 10 min at 2,147 × g (RCF) to separate the blood serum for the biochemical examination. A spectrophotometer T80+ UV/VIS was used to examine the extracted serum (Bioanalytical Diagnostic Industry, Co.). During the experiments, a number of biochemical variables were tracked, including (glucose, total protein, albumin, globulin, A/G ratio, liver function, and kidney function).

### 2.8. Lead residue

The serum samples were put into the atomic absorption spectroscopy after being diluted in the autosampler in the ratio 1 + 1 with 1% v/v nitric acid containing 0.02% v/v of Cetrimonium chloride (CTAC; Model SensAA G3000, GBC Scientific Equipment Pty Ltd., Dandenong, Victoria 3,175, Australia) (54).

### 2.9. Statistical analysis

Using a one-way analysis of variance, all data were checked for normality (Shapiro–Wilk test) and homogeneity of variances (Levene’s test; 55). Whether the variance was equal or unequal, Fisher’s LSD and Dunnett’s *post hoc* tests were employed to compare the various groups. *p* < 0.05 values were regarded as significantly different.

### 2.10. Ethical statement

Experimental setup and fish handling were approved by the Research Ethics Committee (REC) of the Molecular Biology Research & Studies Institute (MBRSI), Assuit University, Assuit, Egypt (No. IORG0010947-22-2023-0026).

## 3. Results

### 3.1. Hematological indices

Exposing African catfish to Pb displayed a significant decline in RBC count (*p* = 0.015), Hb (*p* = 0.003), Ht (*p* = 0.001), thrombocytes (*p* = 0.000), and lymphocytes (p = 0.000), whereas other differential leukocyte counts [neutrophils (*p* = 0.000), monocytes (*p* = 0.001), and eosinophils (*p* = 0.000)] recorded an increase compared with the fish in the control group as shown in Table 1. Although the fish in the group exposed to MPs exhibited a significant rise in both Ht (*p* = 0.020) and MCV (*p* = 0.010), the interaction of both MPs and Pb for 15 days exhibited a remarkable reduction in the fish RBCs (*p* = 0.033), thrombocytes (*p* = 0.009), while differential leukocyte counts [neutrophils (*p* = 0.000), monocytes (*p* = 0.015) and eosinophils (*p* = 0.017)] showed an increase compared to the control fish. Leukocytes’ count (WBCs) reported no change among the experimental groups (0.878 ≤ *p* ≥ 0.130; Table 1).

**Table 1:** Single and combined effect of Pb and MPs on the hematological parameters of African catfish (*Clarias gariepinus*).

	Control	Pb	MPs	MPs + Pb
(RBC’s; ×10^6^/mm^3^)	3.2 ± 0.04^a^	3 ± 0.1^b^	3.02 ± 0.04^ab^	3 ± 0.1^b^
Hemoglobin (Hb; g/dL)	9 ± 0.14^a^	8 ± 0.3^b^	9 ± 0.3^a^	8.1 ± 0.1^ab^
Ht (PCV; %)	35.4 ± 0.1^a^	33.3 ± 0.7^b^	37 ± 0.2^c^	35.3 ± 0.1^a^
MCV (μm^3^)	116 ± 2^a^	112 ± 3^a^	121 ± 1.8^b^	117 ± 2.2^ab^
MCH (Pg)	28.3 ± 0.3^a^	26 ± 0.8^b^	29 ± 0.9^a^	28 ± 0.5^ab^
MCHC (%)	25.4 ± 0.5^a^	23.1 ± 1.1^a^	24 ± 0.7^a^	24 ± 0.5^a^
Thrombocytes (×10^3^/mm^3^)	213 ± 0.5^a^	200 ± 0.9^b^	217 ± 2.8^a^	206 ± 1.3^c^
(WBC’s; ×10^3^/mm^3^)	11 ± 0.2^a^	11.1 ± 0.3^a^	11 ± 0.3^a^	10.5 ± 0.2^a^
Neutrophils (%)	11 ± 0.3^a^	16 ± 0.3^b^	11.3 ± 0.3^a^	14 ± 0.3^c^
Lymphocyte (%)	85 ± 0.5^a^	76 ± 0.3^b^	84 ± 0^a^	80 ± 0.3^c^
Monocyte (%)	3 ± 0.3^a^	4.3 ± 0.2^b^	3 ± 0.3^a^	4 ± 0.3^b^
Eosinophils (%)	2 ± 0^a^	5 ± 0.3^b^	2 ± 0.0^a^	3 ± 0.3^c^

Data are represented as means ± SE. Values with different superscript letters in the same row for each parameter are significantly different (*P* < 0.05).

### 3.2. Inflammatory signals (cytokines)

**Figure 1:**
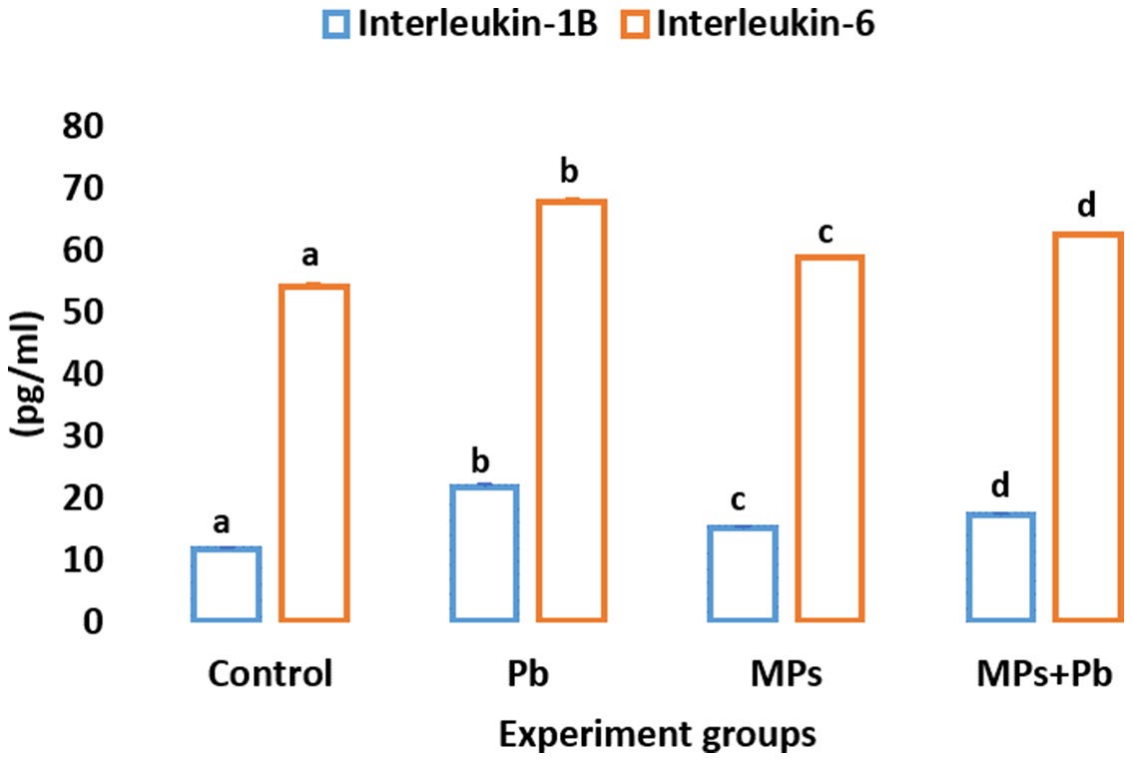
Single and combined effect of Pb and MPs on the immunological parameters of African catfish (*Clarias gariepinus*).

The fish exposed to single and combined treatment of MPs and Pb showed a significant enhancement (*p* = 0.000, *p* = 0.000, respectively) in serum IL-1β and IL-6 cytokines by 1.8, 1.2, 1.4, and 1.2, 1.0, 1.1 folds, respectively, as compared to the control fish. Among the treatment groups, the highest levels of both IL-1β and IL-6 were displayed in the fish exposed to lead (Figure 1).

### 3.3. Electrolyte balance parameters

Plasma electrolytes including Na^+^, Fe^+2^, and Ca^+2^ were increased markedly (*p* = 0.002, *p* = 0.036, *p* = 0.001, respectively) following 15 days of Pb exposure of the fish. Meanwhile, the levels of Na^+^, K^+^, and Fe^+2^ remained unchanged in the fish exposed to MPs (*p* = 0.091, *p* = 0.866, *p* = 0.850, respectively). The fish exposed to both MPs and Pb recorded a significant alteration (Na^+^:*p* = 0.012, Fe^+2^: *p* = 0.022, Ca^+2^: *p* = 0.000, K^+^: *p* = 0.041, HCO_3_^−^: *p* = 0.000, Cl^−^: *p* = 0.024) overall the plasma electrolytes. However, the anion gap showed non-significant differences (0.807 ≤ *p* ≥ 0.074) among the experiment groups (Table 2).

**Table 2:** Single and combined effect of MPs and Pb on the electrolyte balance parameters of African catfish (*Clarias gariepinus*).

	Control	Pb	MPs	MPs + Pb
HCO_3_(μg/mL)	19.8 ± 1.1^a^	18 ± 0.4^a^	14.5 ± 0.3^b^	13.3 ± 0.5^b^
Na^+^ (μg/mL)	126 ± 1.5^a^	138 ± 3.9^b^	120 ± 1.3^ac^	117 ± 0.5^c^
K^+^ (μg/mL)	4.1 ± 0.1^a^	3.7 ± 0.1^b^	4.1 ± 0.1^a^	4.4 ± 0.1^c^
Cl^−^ (μg/mL)	93 ± 2.6^a^	99 ± 1.8^a^	82 ± 3.3^b^	84 ± 2.1^b^
Fe^+2^ (μg/mL)	15.5 ± 0.4^a^	17 ± 0.6^b^	15.6 ± 0.5^a^	17.2 ± 0.4^b^
Ca^+2^ (μg/mL)	51.4 ± 0.7^a^	55 ± 0.6^b^	49 ± 0.5^c^	47 ± 0.4^d^
Anion gap	14 ± 2.8^a^	22 ± 5^a^	24.2 ± 4^a^	20.4 ± 2.6^a^

Data are represented as means ± SE. Values with different superscript letters in the same row for each parameter are significantly different (*P* < 0.05).

### 3.4. Antioxidant parameters and lipid peroxidation

**Figure 2:**
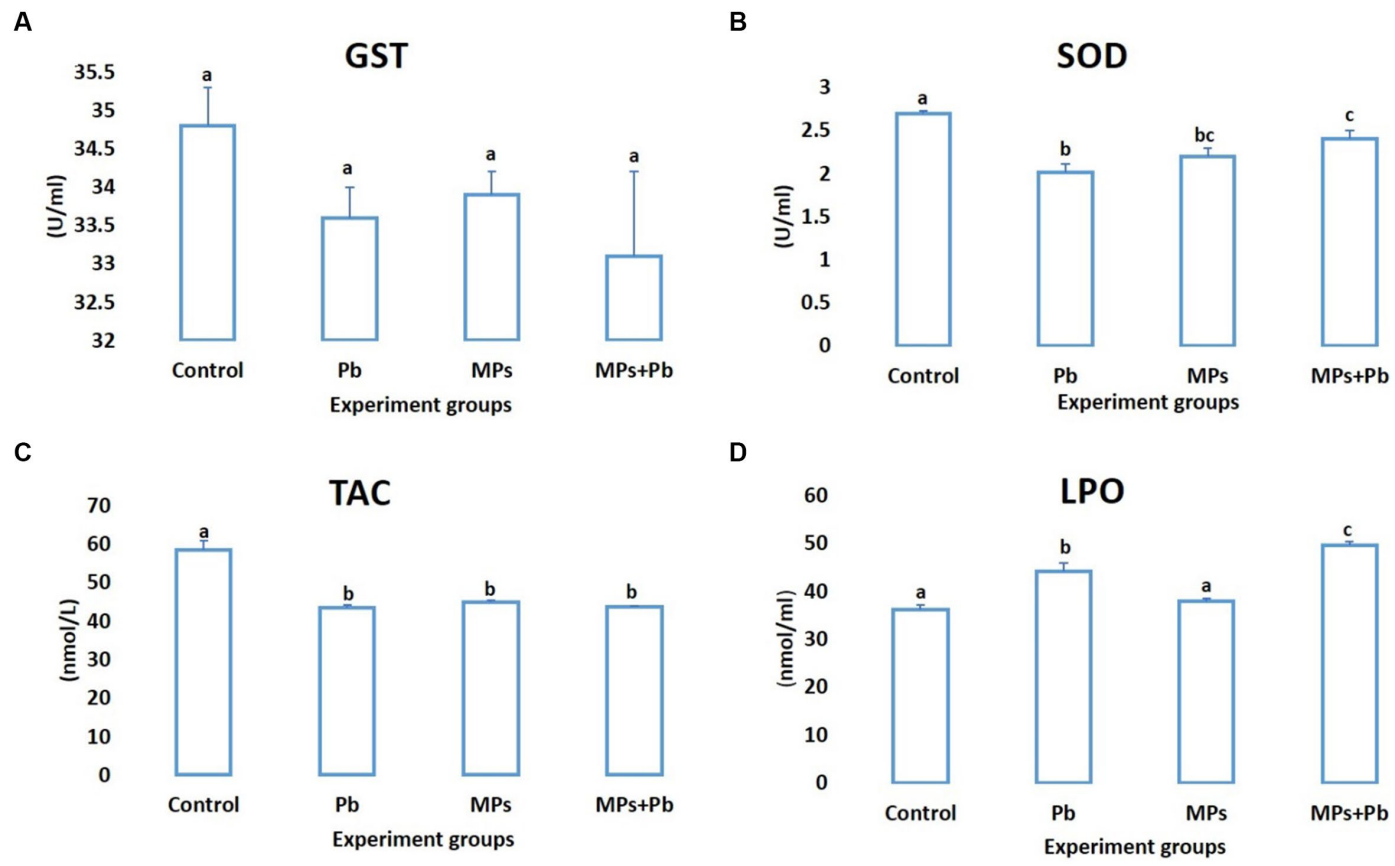
Single and combined effect of MPs and Pb on **(A)** GST, **(B)** SOD, **(C)** TAC, and **(D)** LPO of African catfish (*Clarias gariepinus*).

Compared to the control fish, the levels of SOD and TAC exhibited a notable decline (*p* = 0.005 and *p* = 0.000, respectively) in the fish subjected to individual and combined treatment of MPs and Pb by 25%, 18%, 11%, and 25%, 23%, 25%, respectively. While lipid peroxidation showed a significant increase in lead (*p* = 0.000) and the combination group (MPs and lead; *p* = 0.000). The activity of GST also recorded a non-significant reduction (0.198 ≤ *p* ≥ 0.081) among the experiment groups (Figure 2).

### 3.5. Biochemical parameters

Table 3 shows the effect of Pb, MPs, and their combined effect on the biochemical indices of African catfish for 15 days. Total protein levels recorded a remarkable elevation (*p* = 0.000) in the fish subjected to individual and combined treatment of MPs and Pb compared to the control group. AST showed a significant increase in the combination group compared to the remaining groups (*p* = 0.001), while ALT showed non-significant changes during the study (0.654 ≤ *p* ≥ 0.088) compared to the control group. The other biochemical indices displayed a significant alteration (*p* = 0.000) in the fish exposed to the combined effect of MPs and Pb.

**Table 3:** Single and combined effect of MPs and Pb on the biochemical parameters of African catfish (*Clarias gariepinus*).

	Control	Pb	MPs	MPs + Pb
ALT (μ/L)	17.4 ± 0.4^a^	16.9 ± 0.2^a^	17.5 ± 0.9^a^	19.8 ± 1.4^a^
AST (μ/L)	34 ± 0.4^a^	33.2 ± 0.3^a^	33.9 ± 0.3^a^	36.6 ± 0.5^b^
Glucose (mg/dl)	90.7 ± 1.6^a^	75.4 ± 0.5^b^	81.6 ± 3.7^ab^	100 ± 0.8^c^
Creatinine (mg/dL)	0.35 ± 0.01^a^	0.34 ± 0.01^a^	0.36 ± 0.01^a^	0.47 ± 0.01^b^
Urea (mmol/L)	22.6 ± 0.4^a^	22.9 ± 0.3^ab^	23.8 ± 0.2^ab^	24.3 ± 0.8^b^
Total protein (mg/dL)	3.4 ± 0.1^a^	4.5 ± 0.04^b^	4.2 ± 0^.^04^c^	4.3 ± 0.02^c^
Albumin (mg/dL)	1.7 ± 0.1^a^	1.5 ± 0.1^a^	1.5 ± 0.1^a^	1.1 ± 0.0^b^
Globulin (g/dL)	3.2 ± 0.03^a^	3.2 ± 0.1^a^	2.6 ± 0.1^b^	2.4 ± 0.1^b^
A\G ratio	0.51 ± 0.02^ab^	0.46 ± 0.02^ac^	0.57 ± 0.03^b^	0.43 ± 0.02^c^

Data are represented as means ± SE. Values with different superscript letters in the same row for each parameter are significantly different (*P* < 0.05).

### 3.6. Pb concentration in blood serum

**Figure 3:**
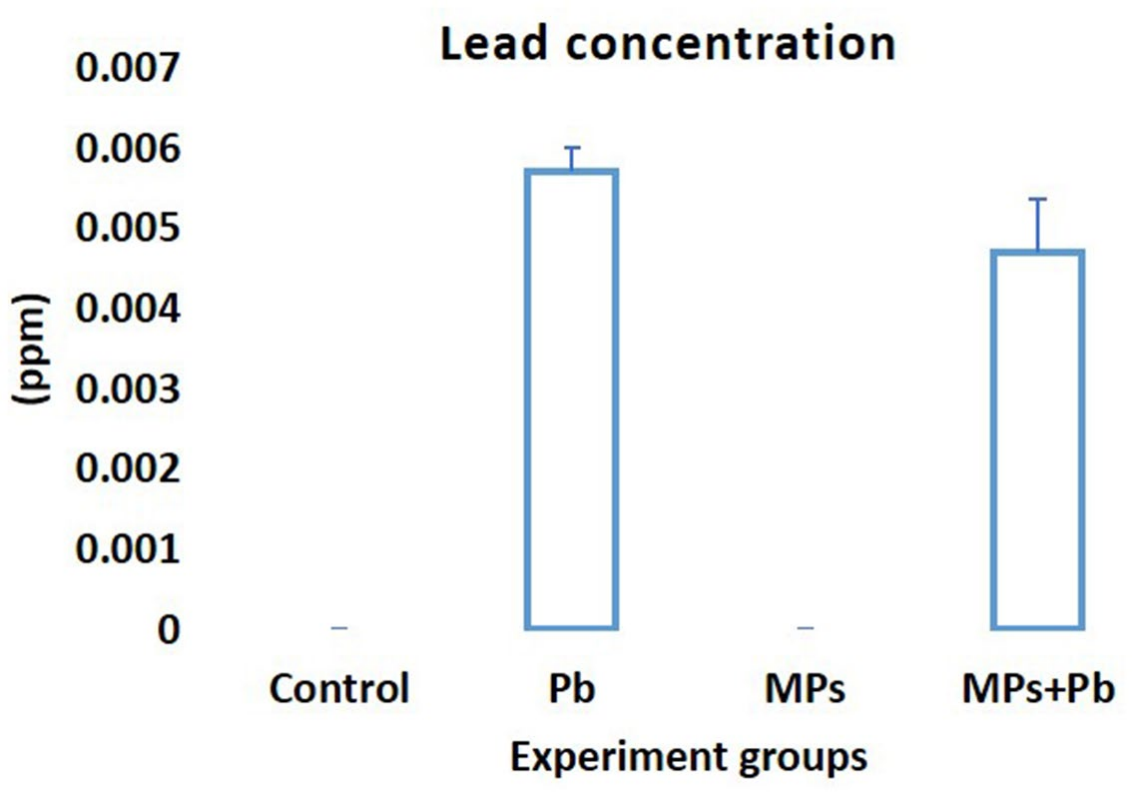
Lead (Pb) concentration (mg/L or ppm) in the blood of African catfish (*Clarias gariepinus*) exposed to the single and combined effect of MPs and Pb.

When compared to the fish exposed to MPs separately or as part of a control group, the mean concentration of Pb (mg/L) in the serum of the fish exposed to Pb alone and Pb/MPs combination significantly increased (*p* = 0.000; Figure 3). The difference between the fish exposed to Pb alone and the fish subjected to Pb and MPs combination was not statistically significant (1.00 ≤ *p* ≥ 0.094; Figure 3).

## 4. Discussion

The present study reported an adverse health effect in African catfish induced by the exposure of Pb and MPs individually or combined. In freshwater, fish are exposed to different types of pollutants at once. Out of all the documented water contaminants, heavy metals are the greatest global threat to fish health and food safety (56). Microplastics are emerging environmental pollutants. Yet, the impact of the combination of heavy metals and MPs on freshwater fish has not been well considered.

Hematological parameters are important in fish health status markers under stressful circumstances (24). RBCs are delicate blood constituents, and several structural and morphological abnormalities may be related to the cellular penetration of metal (57). The exposure of Pb individually or combined with MPs displayed anemia in African catfish. This may be due to hemolysis, dilution of heme, structural change in RBCs, and Hb synthesizing enzymes inhibition or impaired utilization of iron for erythropoiesis (58). In the present study, in comparison to control fish, fish subjected to MPs had considerably lesser levels of MCH, MCHC, and Hb, which confirm an anemic condition. Regarding the increased levels of Fe + in serum, hypochromic microcytic anemia is assured. MCHC is used to indicate the swelling of RBCs (59). The influx of immature RBCs with altered Hb concentration into the circulation can disturb the MCHC and reduce Hb concentration in blood serum (60). The present findings additionally support the hypothesis that hemodilution is a possible reason for Hb content decline in fish exposed to Pb. Similar findings were cited in fish subjected to metals (61, 62). On the other hand, the Fe abundance in the blood of fish exposed to MPs can enhance Fenton’s reaction, which generates more free radicals via the oxidation of ferrous into ferric (63). Heavy metals and MPs exposure caused a significant reduction in the haematological indices in many fish species (16, 18, 20, 22). Although, the WBCs showed no change among the treated groups, the neutrophil-to-lymphocyte ratio of fish exposed to Pb individually or combined with MPs reported a significant increase, indicating the progression of systemic inflammation and immunological responses of fish (64).

Both lymphocyte and neutrophil count variations indicate immunological changes in fish (30). While immunosuppression effects are manifested by high levels of anti-inflammatory cytokines and lymphocyte apoptosis. Here, lymphopenia occurs as a continuous influx of immature neutrophils into the blood (65), hence, our study anticipates that fish exposed to Pb and MPs suffer from tissue damage, which is characterized by increased counts of neutrophils and the release of pro-inflammatory cytokines, oxidizing enzymes, and reactive oxidative species (ROS; 66). The pro-inflammatory cytokines IL-1β and IL-6 are specific for inflammatory responses and several cellular processes [including homeostasis, and cell development, and correlated to autologous immune metabolism (67)]. Both Pb and MPs exposure induced homeostasis disturbance and pro-inflammatory damage in African catfish expressed by enhanced levels of serum cytokines, which is a typical immune response in inflammation and cellular damage (68). Once the cellular homeostasis is interrupted by diseases or tissue damage, interleukins trigger immediate immune responses against this emergent stressor. However, excessive interleukin synthesis has pathological consequences such as serious systemic inflammatory response and immune diseases (69). Wang et al. (70) documented similar inflammatory indications, verified by a higher level of IL-1β expression in hybrid snakehead fish exposed to Nano\MPs and cadmium.

In fish, the concentrations of electrolytes in the bodily fluid are necessary for the dynamic regulation of water inflow and ion outflow. The electrolyte imbalance examines the overall health of fish and serves as a bio-indicator of environmental stresses (23). Contaminants have a toxic effect on the fish gills’ structure and permeability and alter the osmoregulatory dysfunction (71). African catfish exposed to Pb and MPs individually or combined recorded an electrolyte balance disturbance. Previous studies reported the same disturbance of serum electrolytes in fish exposed to metals (72, 73). McCarty and Houston (74) documented that lower levels of Na^+^ and Cl^−^ in plasma were associated with higher levels in fish tissue. Therefore, the present enhancement of serum electrolytes may be related to lower levels in fish tissues, by means of compensation to reduce the stress of Pb. On the other hand, plasma Na^+^ and Cl^−^ ions in African catfish exposed to MPs individually or combined with Pb reported a significant drop. Lower levels of Na^+^ may point to higher epithelial permeability, which disturbs the ion exchange in gills; increase the loss of Na^+^ in the water with inhibition of Na^+^ uptake (75, 76). Regarding our biochemical alterations, renal function impairment may be involved in Na^+^ loss (77). The observed decrease in plasma K^+^ levels may be caused by the gill region’s Pb-sulfhydryl group binding, which can inhibit the ATPase (78). ATPase has a crucial role in ion homeostasis in the gills, while pollutants have a disturbing effect on the ATPase system and osmoregulation in fish (79). The current elevation of plasma K^+^ levels in the combination group could possibly be due to the destruction of erythrocytes which manifested a lower RBC count in MP exposure groups, which likely caused the discharge of K^+^ (80). Generally, higher Ca^+2^ levels can trigger the production of xanthine oxidase and phospholipase enzymes, which promote the production of both the superoxide anion and peroxide radical (81). Consequently, higher Ca^+2^ levels persuaded by Pb and MPs exposure may play a role in the oxidative stress demonstrated in African catfish. Prakash and Verma (73) documented alterations in electrolytes level besides several physiological impairments in fish exposed to MPs. Different sizes and shapes of MPs have induced tissue injuries, inflammation, organ dysfunction, and metabolic alteration in exposed fish (82).

Both SOD and GST activities were reduced along with the total antioxidant capacity (TAC) in the treated groups compared to the control group, especially in the Pb-exposed groups, signifying a disturbance in the redox homeostasis. The antioxidant system is deranged and fails to suppress the emerging oxidative stress and the allied oxidative damage. The antioxidant defense system plays a significant part in the response of fish to different stressors (83). SOD is a key antioxidant enzyme in eliminating excess ROS; it converts superoxide radicals into hydrogen peroxide (84). Xenobiotic and endobiotic metabolite removal depends heavily on glutathione S-transferases (GST). Increased GST activity is a preventive mechanism against oxidative stress and other negative effects when metals enter an organism (85). According to Srikanth et al. (86), even a non-significant increment of ROS in fish exposed to metals could induce serious injuries. Total antioxidant capacity (TAC) is a reliable indicator mirroring the general status of the antioxidant system (including enzymatic and non-enzymatic antioxidants) (87). In fish, MPs exposure can provoke the over-production of reactive oxygen species (ROS), which triggers the antioxidant system and begins to cause cellular damage (84). Metal exposure also stimulates a cellular preventive mechanism against the associated oxidative stress and other negative consequences via the activation of the enzymatic antioxidant system (56). Moreover, MPs-heavy metal complexes were found to alter the antioxidant capacity and cause cellular oxidative damage in fish, while the severity of the oxidative damage is correlated to MPs. Fish have divergent responses against MPs, metal, or both, highlighting the contribution of many physiological processes, which can impair redox hemostasis, cause oxidative stress, and cellular damages (11, 34, 35). However, more research is needed in order to understand these inconsistent responses and the processes involved in the antioxidant system response to metals and MPs.

In consonance with these findings, the present study documented that exposure to Pb and MPs for 2 weeks led to biochemical alterations in African catfish, which may be assigned to their direct noxious effects. The physiological indices that assess the function of organs of African catfish such as; urea, AST, glucose, and total protein displayed a significant enhancement after exposure to Pb and/ or MPs for 2 weeks. Similar variations were detected in fish exposed to MPs and/or nickel (88). Both ALT and AST are significant indexes of liver function. In our study, the combination MPs + Pb exposure group exhibited higher serum AST levels, indicative of hepatic damage concurrently with the recorded oxidative stress (89). Generally, blood carbohydrates and proteins may be metabolized for energy in stress, leading to a significant decline in them (31). Carbohydrate metabolism is associated with advanced levels of blood glucose due to elevated catecholamine levels, which stimulates hepatic gluconeogenesis and glycogenolysis. Hence, higher levels of blood glucose could be a compensatory mechanism, where glucose is used as a source of energy to nullify the toxic impacts of Pb and MPs (89). Enhanced levels of serum urea in African catfish challenged by combination (MPs and Pb) is another evidence of energy intake deficiency, indicating higher amino acids’ gluconeogenesis in the liver that subsequently increases serum ammonia which eventually converted into urea (90). Reduced albumin, globulin, and A/G ratio may refer to amino acid malabsorption in the fish exposed to Pb + MPs, due to oxidative or physical damage to the intestinal lining cells. The function and structure of proteins also could be altered due to the contact of MPs (91). Fish exposed to MPs recorded similar reductions in serum total protein, albumin, and globulin (10).

Moreover, through the detoxification and filtration process to eliminate toxins from the body, Pb was reported to cause kidney and liver dysfunction in fish. In line with our findings, Rahman et al. (92) reported kidney dysfunction and reduced protein synthesis in Nile tilapia exposed to Pb toxicity. Akturk et al. (93) also documented kidney denaturation (inferior glomerular filtration activity and urea excretion capacity) in fish exposed to Pb toxicity.

Among the hazardous effects of MPs on aquatic animals, MPs have reported a strong affinity for adsorbing heavy metals (94). Thus, MPs are considered as an alternative path for the accumulation and transport of heavy metals from the environment to the organisms. More consideration should be given to these new pollutants due to the toxicity of heavy metals and the effects MPs have on the environment. The surface characteristics of MPs allow the direct adsorption of metal ions via the charged sites or neutral regions to form complexes (12). Although the fish exposed to MPs combined with metals reported many physiological and histological alterations (20, 46), there is an ongoing debate about the possible effects of MPs on heavy metals accumulation and toxicity. Contrary to what was anticipated, the accumulation of Pb in fish exposed to a combination (Pb and MPs) was lower than in fish exposed to Pb individually (not statistically significant). In accordance with our result, the accumulation of cadmium in fish exposed to a combination (cadmium and MPs) was lower than in fish exposed to cadmium individually in discus fish, *Symphysodonae quifasciatus* (31). MPs particles can aggregate upon contact with pollutants such as metals, forming larger particle sizes with smaller surfaces and fewer adsorption sites, limiting the bioavailability and toxicity of metals to the challenged organisms (95). According to Zeng et al. (96), the fish exposed to MPs combined with heavy metals has recorded a significant reduction in MPs accumulation in the fish’s gills, as compared to fish challenged with MPs individually. MP particles showed a self-adherent and aggregation behavior when combined with heavy metals on the gill filaments. However, the fish’s body increases mucus secretion through the gills when it detects the physiological changes induced by MPs with heavy metals accumulation in the gills. This response controls the gills’ pressure to expel both MPs and heavy metals from the body (97). Moreover, the absorption of heavy metals on MPs surface involves several forces, including the π-π interactions, oxygen-containing functional groups, and hydrogen bonding which decrease the bioavailability of heavy metals when digested (98). Ingestion of MPs has alleviated the toxicity of heavy metals by absorption, which lowers the bioavailability and accumulation of heavy metals in earthworm gut (99). Moreover, lower Pb in serum may be related to the large molecular size of aggregated combination (MPs and Pb) could be indigestible to fish. Kim et al. (100) reported that among different sizes of polyethylene MPs, fish ingested a limited size range. Ingestion of MPs poses serious physical and/or chemical damage to fish due to their small size. The physical damage depends on the size and aggregation of MPs, while the chemical damage is caused by the polymer additives and absorbed pollutants released inside the fish (101). MPs can interrupt digestion and energy balance in fish, along with affecting nutrient uptake (102). The reported lower accumulation of Pb and energy deficit in African catfish exposed to combination (MPs and Pb) give rise to malnutrition due to the physical damage or blockage of the digestive tract caused by combination MPs + Pb aggregation. This is the primary physical effect of relatively larger MP particles (103).

One of the drawbacks is that lead concentrations in numerous organs, including the muscles, liver, and kidneys, are not measured. Additionally, there is no accepted way of determining the level of microplastics in earlier organs. We anticipate opportunities to assess the concentrations of different elements with microplastics in other fish species (e.g., Tilapia, Zebrafish, Carp, etc.,) or use different metals (e.g., Hg, Cu, Cd, etc.,) because each species of fish or metals can react or behave in a different way which gives different results. For example, In zebrafish, *Danio rerio,* polystyrene (PS) MPs promote cadmium accumulation (30); however, they have the reverse impact in discus fish (*Symphysodonae quifasciatus*) (31).

## 5. Conclusion

Generally, the present study emphasizes the effects of sub-toxic doses of Pb and MPs individually and combined for 15 days on African catfish. The hematological and biochemical alterations along with oxidative stress were recorded in fish. MPs increased the physiological and cytotoxic effects of Pb, although MPs alleviated the accumulation of Pb in the serum of African catfish. So, we anticipate that fish are exposed to the physical damage of MPs more than the chemical damage when combined with Pb.

## Data availability statement

The raw data supporting the conclusions of this article will be made available by the authors, without undue reservation.

## Ethics statement

The animal study was approved by Molecular Biology Research and Studies Institute at Assiut University. The study was conducted in accordance with the local legislation and institutional requirements.

## Author contributions

HS: Conceptualization, Data curation, Investigation, Methodology, Software, Supervision, Writing – original draft, Writing – review & editing. SS: Writing – original draft, Writing – review & editing. MH: Data curation, Investigation, Methodology, Software, Writing – original draft, Writing – review & editing, Conceptualization. AS: Conceptualization, Data curation, Formal analysis, Investigation, Methodology, Software, Supervision, Writing – original draft, Writing – review & editing.
